# Persistent disparities in cholesterol screening among immigrants to the United States

**DOI:** 10.1186/1475-9276-11-22

**Published:** 2012-04-30

**Authors:** Jim P Stimpson, Fernando A Wilson, Rosenda Murillo, Jose A Pagan

**Affiliations:** 1 University of Nebraska Medical Center, Omaha, NE, USA; 2 University of North Texas Health Science Center, Fort Worth, TX, USA; 3 Indiana University, Bloomington, IN, USA

**Keywords:** Immigrants, Screening, Disparities, Cholesterol, Ethnic groups

## Abstract

**Background:**

This study compared differences in cholesterol screening among immigrant populations and US born race/ethnic groups and whether improving access to health care reduced differences in screening.

**Methods:**

Self-reported cholesterol screening for adults was calculated from multivariate logistic regression analysis of the 1988–2008 National Health and Nutrition Examination Surveys (N = 17,118). Immigrant populations were classified by place of birth and length of residency.

**Results:**

After adjusting for individual characteristics and access to health care, the multivariate adjusted probability of cholesterol screening is significantly lower for persons originating from Mexico (70.9%) compared to persons born in the US (80.1%) or compared to US born Hispanic persons (77.8%). Adjustment for access to care did significantly reduce the difference in screening rates between immigrants and natives because the rate for natives remained the same, but the rate for immigrants improved. For example, the difference in screening between US born persons and persons born in Mexico was reduced by nearly 10% after adjustment for access to care.

**Conclusions:**

There are persistent disparities in cholesterol screening for immigrants, particularly recent immigrants from Mexico, but improved access to health care may be a viable policy intervention to reduce disparities.

## Introduction

Although trends in cholesterol have followed a downward trajectory over time, disparities in cholesterol levels persist [1-4]. Theory suggests that the persistence of disparities in cholesterol over time is expected and may be related to changes in prevention and treatment [5]. By studying how cholesterol prevention and treatment efforts vary over time across disadvantaged populations, it may provide insight into disparities for chronic diseases [6,7]. Most research on the disparities of cardiovascular disease risk factors such as cholesterol has been focused on socioeconomic status and race/ethnicity [1]. Nativity status has received less attention in the cholesterol literature despite evidence that it is an important factor differentiating health and limiting access to care [8-13]. Specifically, one study identified that length of residence for immigrants was associated with a higher risk of hyperlipidemia [9]. Another study examined cardiovascular screening practices by nativity and found that persons born in the United States are more likely to be screened for blood pressure and cholesterol than immigrants [10].

With only one published study to indicate whether there is variation by nativity status in screening for high cholesterol levels, there is a need for further information about screening of immigrants. This information is especially needed given the growing research literature documenting barriers to health care for immigrants [12,13]. Therefore, the purpose of this study is to compare differences in cholesterol screening among immigrant populations and US born race/ethnic groups in the US and determine whether improving access to health care would reduce or eliminate the difference.

## Methodology

This study analyzed publicly available data from 1988–2008 provided by the National Center for Health Statistics. The National Health and Nutrition Examination Surveys (NHANES) are nationally representative, cross-sectional surveys that use a stratified, multistage cluster, probability sample design: NHANES III (1988–94), NHANES 1999–2004, NHANES 2005–2008. NHANES is a well-established source of data for health behaviors. The data from these surveys was collected through two methods, the home interview and the health examination. Response rates for examination participants have exceeded 90% since 1988. Additional information can be found within the NHANES documentation [14].

### Measurement

Cholesterol screening was measured by self-reported affirmative response to the following question: “Have you ever had your blood cholesterol checked?”. Nativity was the primary predictor variable and measured by respondent self-reported place of birth. The variable was defined as born in the United States versus not born in the United States. In addition, foreign born respondents were asked the country of origin, which was defined as Mexico versus other country of origin. Length of residence in the US was defined as < 5 years, 5–14 years, and 15 or more years. Race/ethnicity for persons born in the United States was assessed as non-Hispanic White, non-Hispanic African American, Hispanic/Latino, and other race/ethnicity status. Factors that might confound the relationship between nativity and cholesterol screening were also measured including age, sex, education (< high school, high school, > high school), health insurance, and usual source of health care.

### Statistical analysis

All analyses were performed with STATA SE/11 (StataCorp, College Station, TX) to adjust for the complex sampling design of NHANES. For the purposes of this study the analysis only included adults 20–74 years of age that did not report current pregnancy with complete information on all study variables. The final analysis sample size was 17,118 persons. The trend analyses from 1988–2008 are adjusted for survey design, age, and sex. All logistic regression coefficients were converted into probabilities to ease interpretation by using the survey weighted means of the control variables. Year of age squared was used in the analyses to capture the non-linear relationship between age and screening. Percentages in all analyses were adjusted to the 2007–2008 NHANES demographic distribution. The multivariate regression pooled data from 1999–2008 to allow for larger sample sizes of foreign born persons which would increase statistical variation. As indicated in the previous literature, access to health care may influence whether immigrants receive cholesterol screening [8-13]. Therefore the regression results are calculated in two steps. The first regression model adjusts for sample weight, cluster effects, survey year, age and its square, sex, race/ethnicity, and education. The second regression model adds adjustments for insurance status and usual source of care.

## Results

Table 1 shows the trend in prevalence for cholesterol screening. There are statistically significant differences between US natives and Mexican immigrants from 1988–2008. After increasing from 35.5 to 51.9% in 1988–2004, the likelihood of screening for Mexican immigrants failed to improve after 2004, increasing the disparity in screening between Mexican born and US born persons to a 33.8% point difference by 2005–08. Differences between US born Hispanic persons and Mexican immigrants also increased over time. Screening increased from 52.5 to 76.0% from 1988–2008 for US born Hispanics compared to a smaller increase from 35.5 to 48.8% for Mexican immigrants over this period. Persons originating from Mexico were consistently and significantly less likely to be screened over time than persons born in a country other than Mexico. In 2005–08, the likelihood of Mexican immigrants being screened was only 48.8% vs. 76.7% for immigrants from countries other than Mexico. Foreign born persons that have resided in the US five years or more were significantly more likely to be screened than persons that had lived in the US for less time in 2005–08. For example, 31.1% of recent Mexican immigrants had cholesterol screening compared to 52.1% of longer-term immigrants, a difference of 67.5%. Recent other immigrants were also significantly less likely to screen than other immigrants with at least five years of residency.

**Table 1:** Age and Sex Adjusted Prevalence of Cholesterol Screening by Country of Origin, Race/ethnicity, and Length of US Residency: National Health and Nutrition Examination Surveys 1988-2008

		1988-94			1999-04			2005-08	
	N	%	95% CI	N	%	95% CI	N	%	95% CI
Born in the United States	10885	66.7	(63.9, 69.6)	8623	79.0	(77.0, 80.9)	6491	82.6	(80.4, 84.9)
White, non-Hispanic	4907	69.7	(66.7, 72.7)	5012	80.7	(78.6, 82.8)	3685	83.7	(81.2, 86.1)
Hispanic/Latino	2057	52.5	(47.5, 57.6)	1294	70.7	(65.2, 76.1)	804	76.0	(72.7, 79.3)
African American, non-Hispanic	3879	57.0	(53.4, 60.7)	2125	71.2	(68.6, 73.8)	1846	78.6	(76.1, 81.2)
Other race/ethnicity	42	44.7	(32.2, 57.2)	192	71.3	(65.3, 77.4)	156	79.1	(73.5, 84.7)
Born in Mexico	1531	35.5	(31.0, 40.0)	1551	51.9	(47.7, 56.1)	1008	48.8	(44.6, 52.9)
*Residence in US*:									
< 5 years	362	24.3	(14.4, 34.2)	311	44.7	(38.0, 51.5)	157	31.1	(21.9, 40.3)
5+ years	1169	37.7	(32.8, 42.6)	1240	53.1	(48.4, 57.8)	851	52.1	(47.9, 56.3)
Born Elsewhere	811	55.3	(48.6, 62.1)	1097	74.5	(70.6, 78.5)	1016	76.7	(72.4, 80.9)
*Residence in US*:									
< 5 years	168	41.9	(29.4, 54.3)	193	68.7	(62.0, 75.5)	136	60.9	(48.9, 72.8)
5+ years	643	57.7	(50.3, 65.0)	904	75.5	(71.4, 79.5)	880	78.9	(75.2, 82.7)

Note: Analyses are restricted to persons 20–74 years of age with complete information on study variables and percentages are adjusted for sample weights, clustering, age and sex.

Table 2 presents the multivariate stepwise-adjusted probabilities for cholesterol screening by country of origin and length of residence from 1999–2008. After adjusting for individual characteristics and access to health care, the multivariate adjusted probability of cholesterol screening is significantly lower for persons originating from Mexico (70.9%) compared to persons born in the US (80.1%) or compared to US born Hispanic persons (77.8%). Length of residence for persons originating from Mexico exacerbates the screening disparity; persons from Mexico that have resided in the US less than five years were 6% less likely to report cholesterol screening compared to persons from Mexico with greater than five years of residency and 12% less likely than US born Hispanics. Persons originating from a country other than Mexico were not significantly different in screening compared to persons from the US regardless of length of residence. Adjustment for access to health care did not alter the probabilities of screening for US natives when compared to the first model. However, adjustment for access to care did increase the probabilities of screening for immigrants. For example, the screening rate for persons born in Mexico jumped from 62% to 71% after adjustment for access to care. The difference in screening rates between immigrants and natives was significantly reduced because the rate for natives remained the same, but the rate for immigrants improved. For example, the difference in screening between US born persons and persons born in Mexico was reduced by nearly 10% after adjustment for access to care.

**Table 2:** Multivariate Adjusted Percentage of Self-Reported Cholesterol Screening by Country of Origin, Race/ethnicity, and Length of US Residency: National Health and Nutrition Examination Survey 1999-2008

		1		2	
	N	%	95% CI	%	95% CI
Born in the United States	14992	80.4	(79.0, 81.7)	80.1	(78.8, 81.5)
White, non-Hispanic	8643	81.3	(79.8, 82.8)	81.0	(79.5, 82.5)
Hispanic/Latino	2071	76.2	(73.0, 79.5)	77.8	(74.4, 81.1)
African American, non-Hispanic	3932	76.7	(75.1, 78.4)	76.6	(74.9, 78.2)
Other race/ethnicity	346	74.2	(70.1, 78.3)	74.3	(70.0, 78.6)
Born in Mexico	2539	62.4	(59.5, 65.4)	70.9	(68.4, 73.5)
< 5 years in US	461	51.6	(45.7, 57.5)	65.4	(59.7, 71.2)
5+ years in US	2078	64.3	(61.1, 67.4)	71.8	(69.1, 74.5)
Born Elsewhere	2086	76.6	(74.1, 79.1)	78.7	(76.4, 81.0)
< 5 years in US	325	67.7	(62.0, 73.4)	74.1	(68.6, 79.6)
5+ years in US	1761	77.9	(75.5, 80.4)	79.4	(77.1, 81.7)

Note: Percentages are calculated from multivariate logistic regression models for persons 20–74 years of age with complete information on study variables. Model 1 adjusts for sample weight, cluster effects, survey year, age and its square, sex, and education. Model 2 adds adjustments for insurance status and usual source of care.

## Discussion

This study set out to determine if screening for cholesterol varied by nativity using nationally representative data. The analysis divided the immigrant population into length of time in the US and country of origin. The results from the paper suggested that these classifications were important for the interpretation of results. Simply showing nativity status without these distinctions may have masked important differences. For example, persons originating from Mexico were often different from persons originating from other countries, including the US. Unfortunately small sample sizes did not allow further classification of country of origin and future studies with sufficient observations should attempt to analyze other countries of origin to further elucidate potential cultural differences in prevention of cholesterol and other health conditions.

The persistent gap in screening over the last 20 years could have been related to efforts aimed at blocking access to health care for immigrants through various health policies [12,13]. In particular, the Personal Responsibility and Work Opportunity Reconciliation Act of 1996 blocked access to government insurance programs for most immigrants until they had lived legally in the United States for five years. Our findings from the age and sex adjusted results suggested that screening differences between immigrants and US born persons worsened after 1999. These findings are consistent with PRWOA reducing access to care among immigrants. However, to test whether the effect of access to care reduced or eliminated the difference in screening rate between immigrants and US natives, we performed a stepped regression analysis. The results from the multivariate findings suggested that access to care reduced, but did not eliminate, the difference in screening rates between immigrants and natives, particularly for immigrants with less than five years of residence. Therefore, improving access to care may be a viable intervention to reduce disparities in cholesterol screening.

The interpretation of our findings should be interpreted within the context that the data are based on self-reported cholesterol screening and represent a cross section of screening disparities. Future research should verify these findings using clinical or administrative sources of data with objective measurement of cholesterol screening and that can track individuals over time. Administrative data may also have larger populations of immigrants to examine screening and prevention of immigrants from countries other than Mexico. Finally, there was still a difference in cholesterol screening that was unexplained. The lower screening rates for immigrants, particularly recent immigrants, could reflect lower perceived need for services, low health literacy, low paid leave, and difficulty in getting an appointment. The data used for this study was unable to account for these factors.

In conclusion, our findings suggest that even after adjustments for numerous factors that might contribute to disparities in screening such as access to a usual source of care and health insurance, there was a persistent disparity in cholesterol screening with persons originating from Mexico, particularly recent immigrants from Mexico, significantly less likely to report screening for cholesterol. However, access to care did significantly reduce differences in screening suggesting that improved access to care may be a viable policy intervention to reduce disparities in chronic diseases. Based on the findings from this study, greater efforts to increase screening should be directed toward immigrant populations, and in particular attend to differences in country of origin.

## Competing interests

The authors declare that they have no competing interests.

## Authors' contributions

JPS designed the study and drafted the manuscript. FAW carried out the statistical analysis and assisted with interpretation of findings. RM assisted with drafting the manuscript and the conceptualization of the study design. JAP assisted with drafting the manuscript and interpretation of finding. All authors read and approved the final manuscript.
